# Initial screening for occult congenital ectopia lentis based on ocular biological parameters in preschool children

**DOI:** 10.1186/s12886-023-03230-7

**Published:** 2023-11-27

**Authors:** Jiaona Jing, Qingwei Meng, Wei Gu, Haixia Cheng, Kun Li, Yuming Li, Qinghuai Liu

**Affiliations:** 1 Department of Ophthalmology, Children’s Hospital of Nanjing Medical University, Nanjing, China; 2 Department of Ophthalmology, The First Affiliated Hospital of Kunming Medical University, Kunming, China; 3 Department of Medical, Children’s Hospital of Nanjing Medical University, Nanjing, China; 4 Harris Laboratory, Boston Children’s Hospital, Harvard Medical School, Boston, USA; 5 Department of Ophthalmology, The First Affiliated Hospital of Nanjing Medical University, Nanjing, China

**Keywords:** Congenital ectopia lentis, Myopia, Biological parameters, Axial length-corneal radius ratio, Preschool children

## Abstract

**Background:**

This study aimed to identify an initial screening tool for congenital ectopia lentis (CEL) by comparing ocular biological parameters in children with myopia.

**Methods:**

A retrospective case-control study was conducted at one tertiary referral centre, from October 2020 to June 2022. Axial length (AL), corneal curvature (CC), refractive astigmatism (RA), corneal astigmatism (CA), internal astigmatism (IA), the difference between the axis of RA and CA [AXIS(RA-CA)], white-to-white corneal diameter (WTW), and axial length-corneal radius ratio (AL/CR) were compared in 28 eyes of CEL patients, and 60 eyes of myopic patients matched for age and refraction. The spherical equivalent of each eye was < -3.00 D. Area under the curve (AUC) of the receiver operating characteristic curves were calculated.

**Results:**

The differences in RA, AL, mean keratometry (Kmed), maximum keratometry (Kmax), minimum keratometry (Kmin), CA, IA, AXIS(RA-CA), WTW, and AL/CR between the CEL and myopic groups were statistically significant (p < 0.05; p < 0.001; p < 0.001; p < 0.001; p < 0.001; p < 0.05; p < 0.001; p < 0.001; p < 0.001; p < 0.001, respectively). In logistic regression analysis RA, IA, AXIS(RA-CA), and AL/CR were significantly associated with CEL (p < 0.05). AUCs for RA, IA, AXIS(RA-CA), and AL/CR were 0.694, 0.853, 0.814, and 0.960, respectively. AUCs for AL/CR in SE< -6.00 D subgroup was 0.970, and 0.990 in -6.00 D ≤ SE < -3.00 D group. An AL/CR < 3.024 was the optimal cut-off point differentiating the CEL and control groups (sensitivity, 92.9%; specificity, 88.30%).

**Conclusions:**

A smaller AL/CR could identify CEL in children with myopia. An AL/CR cut-off value of 3.024 may be the most sensitive and specific parameter for the differential diagnosis of CEL in patients with mild to high myopia.

## Introduction

Ectopia lentis is the dislocation of the lens from its normal position. Ectopia lentis can be categorised as subluxated and luxated, referring to the partial or complete displacement of the lens. Subluxated lens refers to a partial displacement of the lens, with a number of the suspensory ligaments remaining intact so that part of the lens remains in the pupillary area. The classification of ectopia lentis also includes primary (congenital), secondary (consecutive), and traumatic lens dislocations [1]. Congenital ectopia lentis (CEL) is often associated with inherited connective tissue disorders such as Marfan syndrome (MFS), Weill–Marchesani syndrome, homocystinuria, Ehlers–Danlos syndrome, and those without systemic associations [2–5].

CEL can lead to refractive error, marked astigmatism, amblyopia, strabismus, and diplopia, especially during the critical period during ocular development. In addition, CEL is often complicated by eye diseases, such as cataract formation, glaucoma, retinal detachment, and other complications that can lead to permanent loss of vision [6]. Due to the serious complications of CEL, early diagnosis and treatment are extremely important for decreasing the risk of visual impairment and blindness. A previous study showed that the average age at which CEL surgery is performed was 9.24 ± 4.83 years, which means that delayed hospital referrals and late treatment are very common [7]. This may be due to the poor expression of young children and an uncomplete ophthalmic evaluation using the slit lamp or ultrasound biomicroscopy (UBM). Moreover, under non-mydriatic conditions, the atypical clinical symptoms of CEL with slight subluxation and iridodonesis are easily ignored by doctors.

Myopia is a common manifestation of CEL due to lenticular myopia resulting from increased curvature of the lens, and axial myopia caused by increased axial length (AL) [6, 8]. Myopia greater than − 3.00 D is relatively common in children with inherited connective tissue diseases. Previous studies have reported that myopia is the second common ocular manifestation in MFS, in which 16.3% have myopia < -7.00 D, and about 50% have myopia < -3.00 D [9, 10]. The presence of high myopia in homocystinuria patients is also approximately 45% [11]. Therefore, it is very important to screen lens subluxation in young child with myopia < -3.00 D.

In the present studies, UBM was frequently used to diagnose ectopia lentis [12, 13]. However, the diagnosis of CEL has certain limitations in children. Many studies have shown that AL, corneal astigmatism (CA) and higher-order aberrations also can be influenced by CEL [14–16]. Therefore, this study aimed to discover an efficient and convenient method to distinguish and initially diagnose CEL in children with myopia.

## Methods

### Design

This retrospective study was conducted from October 2020 to June 2022 at the Department of Ophthalmology, Children’s Hospital of Nanjing Medical University, Nanjing, China. The study was conducted in accordance with the Declaration of Helsinki and approved by the Ethical Committee of the Children’s Hospital of Nanjing Medical University. Informed consent was obtained from all parents or guardians.

### Participants

A total of 28 children (28eyes) with CEL and myopia < -3.00 D diagnosed by experienced ophthalmologists. For comparison, a group of 60 children (60eyes) matched in age and refractive error were selected as control. The right eye of each patient was selected for subsequent analyses. All participants were aged 3–7 years. Patients with secondary lens dislocations, such as those with ocular and head trauma, history of ocular surgery, history of corneal disease, or any other ocular disease were excluded from this study. Both eyes of each participant were examined. The criteria for occult CEL, used in this study, was slit lamp examination showing the lens almost located in its normal position in natural pupil state but exhibiting mild subluxation after cycloplegia.

### Eye examinations

Participants underwent thorough ophthalmic examinations by trained technicians who were in charge of examination. The patient’s family and medical histories were evaluated before examination. All participants underwent slit-lamp biomicroscopy (Topcon SL-2G, Topcon Inc., Tokyo, Japan) of the anterior segment by certified ophthalmologists before and after pupillary dilation. Refraction was performed using a Topcon KR-1 autorefractometer (Topcon Inc., Tokyo, Japan) prior to pupillary dilation. AL and white-to-white corneal diameter (WTW) values were measured using an IOL Master 500 (software version V.5.5, Carl Zeiss Meditec AG, Jena, Germany). The maximum and minimum values of corneal curvature (Kmax and Kmin) were measured using the ATLAS corneal topography 9000 (Carl Zeiss Meditec Inc., California, USA). All measurements were repeated three times for each eye, and the average value was recorded.

### Definitions

Refractive errors were estimated with regard to both spherical and cylindrical power (S and C, respectively) and expressed in diopters (D). The spherical equivalent (SE) was used to calculate the average refractive error and was derived from the sum of the spherical power with half of the cylindrical power [$\text{S}+(0.5\times \text{C})$]. Mean keratometry (Kmed) was considered as the mean value of Kmin and Kmax, and CA was calculated as $\text{K}\text{m}\text{a}\text{x}-\text{K}\text{m}\text{i}\text{n}$. Refractive astigmatism (RA) was the total astigmatism, which referred to cylindrical power. Internal astigmatism (IA) was calculated as $\text{R}\text{A}-\text{C}\text{A}$. AXIS(RA-CA) was the difference between the axis of RA and CA. The mean corneal radius (CR) was extracted from the Kmed value using the formula: $\text{C}\text{R} \left(\text{m}\text{m}\right)=1000\times 0.3375/\text{K}\text{m}\text{e}\text{d}\left(\text{D}\right)$. The axial length-corneal radius ratio (AL/CR) was defined as the AL divided by the mean CR of the curvature.

### Statistical analysis

All statistical analysis were performed using SPSS software version 26.0 (IBM Corp., Armonk, NY, USA). Quantitative data with normal distributions are presented as mean ± standard deviation, and differences between groups were compared using Student’s t-test; a non-parametric Wilcoxon rank-sum test was used for skewness distribution data, presented as medians (interquartile range); categorical variables are presented as counts and percentage and were analyzed using the chi-square test. Univariate logistic regression analyses were performed to investigate the associations between various ocular parameters and CEL. Receiver operating characteristic (ROC) curves were calculated to indicate the separation between the CEL and control groups. ROC curves were constructed using SPSS software version 26.0, then the area under the curve (AUC) was calculated and compared by ROC analysis. The optimal cut-off point was calculated using Youden indices. AUC > 0.75 indicated that the classifier provides clinically meaningful discriminative ability [17]. Statistical significance was set at p-value < 0.05.

## Results

### Patient demographics

In this retrospective case-control study, 28 eyes of CEL patients were compared with 60 eyes of myopic patients of the control group. The demographic characteristics of the patients are summarized in Table 1. There were no significant differences in baseline characteristics between the CEL and control groups (p > 0.05).

**Table 1:** Demographic characteristics of patients in each group

Characteristics	CEL	Control	z/χ2	P-value
Subjects(eyes)	28	60		
Age (years)	4.638(4.638 to 5.312)	4.125(4.000 to 4.638)	-1.799	0.072
Gender			0.064	0.800
male	16(57.10%)	36(60.00%)		
female	12(42.90%)	24(40.00%)		
SE (D)	-6.25(-8.719 to -4.688)	-5.75(-7.594 to -4.156)	-0.766	0.444

CEL: congenital ectopia lentis; SE: spherical equivalent; D: diopters

### Ocular biological parameters of CEL and myopic patients

The differences in RA, AL, Kmed, Kmax, Kmin, CA, IA, AXIS(RA-CA), WTW, and AL/CR between the CEL and myopic groups were statistically significant (p < 0.05; p < 0.001; p < 0.001; p < 0.001; p < 0.001; p < 0.05; p < 0.001; p < 0.001; p < 0.001; p < 0.001; respectively) (Table 2). CEL patients had shorter AL, flatter Kmed, Kmax, Kmin, higher RA, IA, lower CA, smaller WTW, AL/CR, and significant axial changes compared with control patients. On further comparison of RA and CA, results showed that RA was higher than CA (-2.875[-5.938 to -1.313] vs. -1.23[-1.95 to -0.485], p < 0.05) in the CEL group, with no significant difference shown between them in the myopic group (-1.75[-2.6875 to -1] vs. -1.93[-2.56 to -1.268], p > 0.05).

**Table 2:** Comparison of ocular biological parameters of CEL and control groups

Parameters	CEL	Control	Z/F-value	P-value
Spherical power (D)	-4.75(-7.063 to -3.063)	-5(-6.688 to -3.500)	-0.637	0.524
RA (D)	-2.875(-5.938 to -1.313)	-1.75(-2.6875 to -1.000)	-2.927	< 0.05*
AL (mm)	22.96(22.165 to 24.100)	24.48(23.843 to 25.120)	-4.22	< 0.001**
Kmed (D)	41.115(39.478 to 42.765)	43.538(42.755 to 44.745)	-4.963	< 0.001**
Kmax (D)	41.625(39.743 to 43.453)	44.730(43.515 to 45.900)	-4.776	< 0.001**
Kmin (D)	40.73(39.160 to 41.953)	42.46(41.963 to 43.538)	-4.691	< 0.001**
CA (D)	-1.23(-1.950 to -0.485)	-1.93(-2.560 to -1.268)	-2.634	< 0.05*
IA (D)	-1.860(-3.650 to -0.500)	0.240(-4.175 to 0.558)	-5.318	< 0.001**
AXIS(RA-CA)(°)	19.000(9.250 to 31.750)	5.000(2.000 to 10.000)	-4.729	< 0.001**
AL/CR	2.822(2.708 to 2.916)	3.137(3.097 to 3.245)	-6.916	< 0.001**
WTW (mm)	11.707 ± 0.239	12.067 ± 0.242	0.411	< 0.001**

CEL: congenital ectopia lentis; RA: refractive astigmatism; AL: axial length; Kmed: mean keratometry; Kmax: maximum keratometry; Kmin: minimum keratometry; CA: corneal astigmatism; IA: internal astigmatism; AXIS(RA-CA): difference between axis of RA and CA; AL/CR: axial length-corneal radius ratio; WTW: white-to-white corneal diameter; D: diopters. * P < 0.05; ** P < 0.001

### Factors associated with CEL

Logistic regression models were used to assess the relationship between the clinical.

characteristics and biometric parameters of the CEL and control groups, and the results are shown in Table 3. The CEL was considered a positive event and without the CEL.

was considered a negative event. We used age and gender as adjusting variables. After conducting univariate analysis, the variables with a P value of less than 0.05 were included in the multivariate analysis using backward selection procedures. In the multivariate models, the AL/CR, RA, IA, and AXIS(RA-CA) were significantly associated with CEL (p < 0.001; p = 0.002; p < 0.001; p = 0.001, respectively).

**Table 3:** Univariate logistic regression models

Variables	Univariate (adjust)		Multivariate	
	OR (95%)	P-value	OR (95%)	P-value
AL/CR	0.980(0.972,0.989)	< 0.001**	0.975(0.959,0.992)	< 0.001**
AL (mm)	0.405(0.248,0.663)	< 0.001**		
Kmed(D)	0.428(0.286,0.639)	< 0.001**		
Kmax(D)	0.507(0.368,0.697)	< 0.001**	0.574(0.263,1.253)	0.163
Kmin(D)	0.382(0.241,0.605)	< 0.001**		
CA(D)	0.52(0.305,0.884)	0.016*		
RA(D)	0.597(0.438,0.814)	0.001*	0.358(0.184,0.695)	0.002*
IA(D)	0.243(0.124,0.476)	< 0.001**	0.246(0.119,0.509)	< 0.001**
AXIS(RA-CA) (°)	1.112(1.054,1.173)	< 0.001**	1.126(1.047,1.211)	0.001*
WTW(mm)	0.002(0.000,0.027)	< 0.001**		

AL/CR: axial length-corneal radius ratio; AL: axial length; SE: spherical equivalent; Kmed: mean keratometry; Kmax: maximum keratometry; Kmin: minimum keratometry; CA: corneal astigmatism; RA: refractive astigmatism; IA: internal astigmatism; AXIS(RA-CA): difference between axis of RA and CA; AL/CR: axial length-corneal radius ratio; WTW: white-to-white corneal diameter; D: diopters. * P < 0.05; ** P < 0.001

### ROC curves of ocular biological parameters for the differential diagnosis of CEL

To explore the potential differential value of biological parameters for CEL diagnosis, the results in Table 3 that showed considerable positive statistical significance between.

**Fig. 1:**
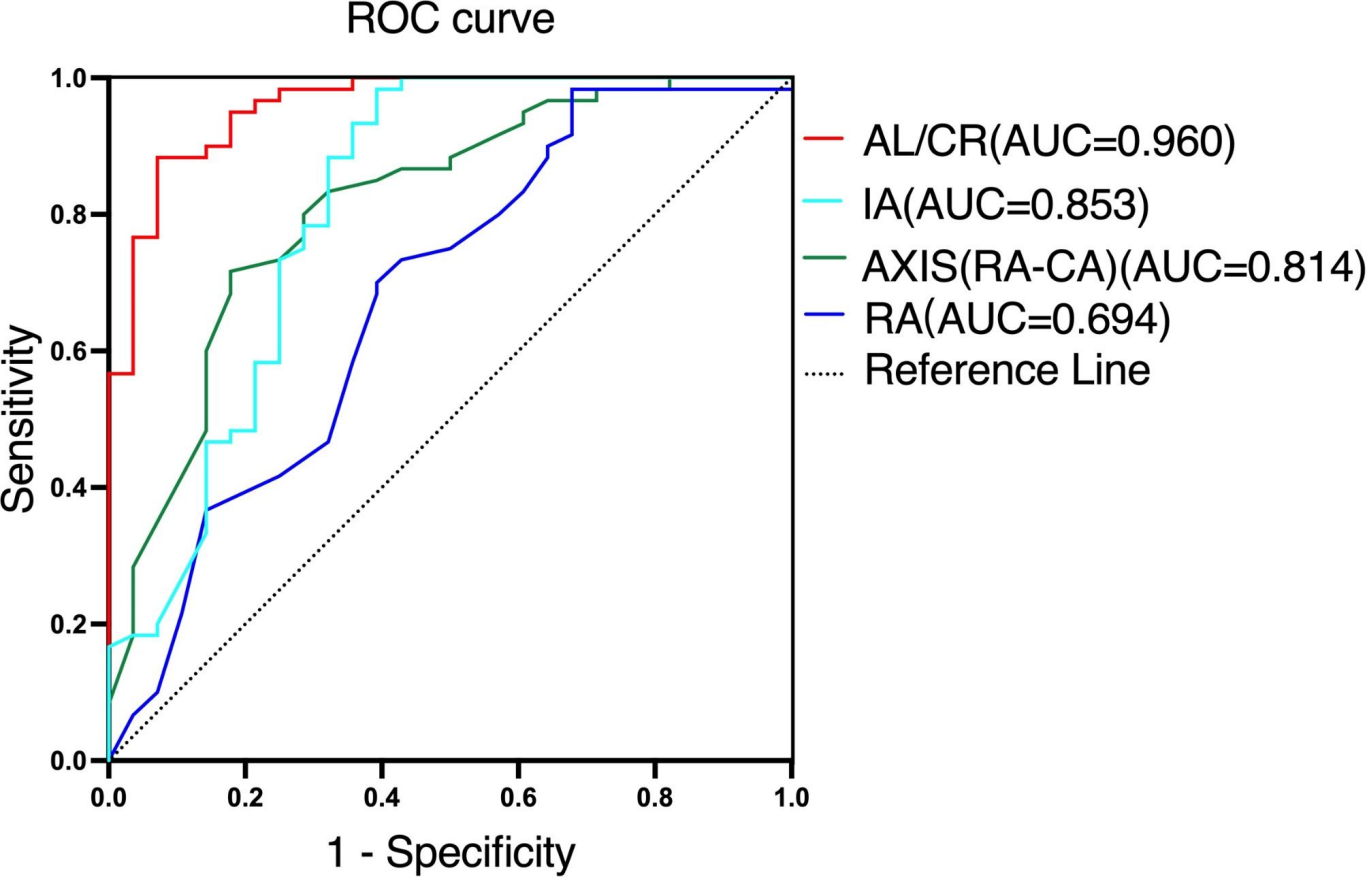
ROC curves of ocular biological parameters for the differential diagnosis of CEL. ROC: receiver-operating characteristic; CEL: congenital ectopia lentis; AL/CR: axial length-corneal radius ratio; IA: internal astigmatism; AXIS(RA-CA): difference between axis of RA and CA; RA: refractive astigmatism; AUC: area under the curve

CEL and biological parameters, were further analyzed using ROC curves. The AUC was 0.960 for AL/CR, 0.694 for RA, 0.853 for IA, and 0.814 for AXIS(RA-CA) (Fig. 1; Table 4). An AL/CR value of 3.024 was found to be the optimal cut-off point between the CEL and control groups, representing a sensitivity of 92.9% and specificity of 88.3% (Table 4).

Although the AUC of RA is less than 0.75, we still evaluated its diagnostic capacity in comparison to AL/CR. AL/CR has a stronger diagnostic ability compared to RA, IA, and AXIS(RA-CA) (p < 0.001). There is no significant difference in diagnostic ability between RA, IA, and AXIS (RA-CA) (p > 0.05).

We further analyzed the AUC for AL/CR in different SE. The AUC was 0.99 with a specificity of 91% in SE < -6.00 D group, and 0.97 with a specificity of 82% in -6.00 D ≤ SE < -3.00 D group (Table 5).

**Table 4:** ROC curve analysis for RA, IA, AXIS(RA-CA), and AL/CR

Parameters	AUC (95%CI)	Cut-off value	Sensitivity	Specificity	P-value
AL/CR	0.960(0.920,0.999)	3.024	92.9%	88.3%	< 0.001**
RA (D)	0.694(0.569,0.819)	-2.375	60.7%	71.7%	< 0.05*
IA (D)	0.853(0.758,0.948)	-0.47	79%	80%	< 0.001**
AXIS(RA-CA) (°)	0.814(0.715,0.912)	8.5	82%	72%	< 0.001**

AL/CR: axial length-corneal radius ratio; RA: refractive astigmatism; IA: internal astigmatism; AXIS(RA-CA): difference between axis of RA and CA; D: diopters. * P < 0.05; ** P < 0.001

**Table 5:** ROC curve analysis for AL/CR in subgroups

	AUC (95%CI)	Cut-off value	Sensitivity	Specificity	P-value
-6.00 D ≤ SE < -3.00 D	0.970(0.931,1)	3.024	100%	82%	< 0.001**
SE < -6.00 D	0.990(0.966,1)	3.13	100%	91%	< 0.001**

AL/CR: axial length-corneal radius ratio; SE: spherical equivalent; D: diopters. ** P < 0.001

### Proportions of ocular biological parameters of CEL and myopic patients

**Fig. 2:**
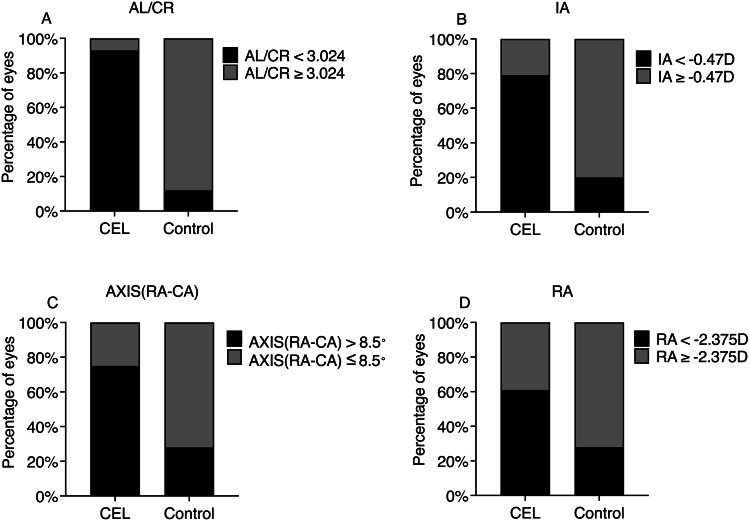
Proportions of ocular biological parameters among CEL and myopic eyes. **A** 93% of CEL eyes had AL/CR less than 3.024 compared to 12% of myopic eyes. **B** 79% of CEL eyes had IA less than -0.47 D, compared to 20% of myopic eyes. **C** 75% of CEL eyes had AXIS (RA-CA) more than 8.5° compared to 28% of myopic eyes. **D** 61% of CEL eyes had RA less than -2.375 D compared to 28% of myopic eyes. AL/CR: axial length-corneal radius ratio; IA: internal astigmatism; AXIS(RA-CA): difference between axis of RA and CA; RA: refractive astigmatism; D: diopters

The maximum values of the Youden index were used as a criterion for selecting the optimal cut-off points and for each of the tested parameters they were as follows:

AL/CR, 3.024; IA, -0.47 D; AXIS (RA-CA), 8.5°; and RA, -2.375 D. 93% of CEL eyes had AL/CR less than 3.024 compared to 12% of myopic eyes (p < 0.001). In the CEL group, 79% of CEL eyes had IA less than -0.47 D, compared to 20% of myopic eyes (p < 0.001), 75% of CEL eyes had AXIS (RA-CA) more than 8.5°, compared to 28% of myopic eyes (p < 0.001). Finally, 61% of CEL eyes had RA less than -2.375 D, compared to 28% of myopic eyes (p < 0.05) (Fig. 2).

## Discussion

**Fig. 3:**
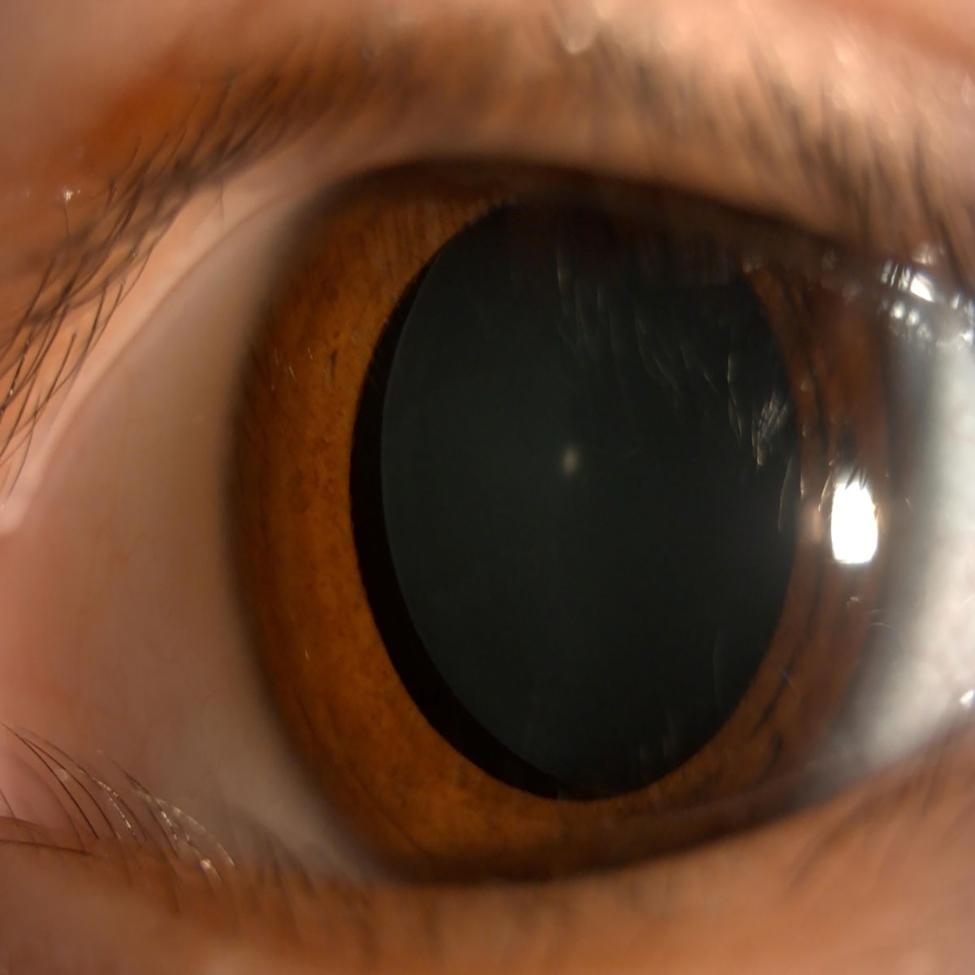
Representative case in the congenital ectopia lentis group. The left eye of a 5-year-old girl with a temporally subluxated lens. The pupil is dilated with compound topicamide. Poor vision in both eyes is observed at her eye examination. Ophthalmic examination reveals a visual acuity of 6/21 in the left eye. Other ophthalmic examinations show mean keratometry of 40.88 D; axial length of 21.89 mm; and axial length-corneal radius ratio of 2.715 in the left eye. Refractive astigmatism is greater than corneal astigmatism in the left eye (-1.50 D and − 0.97 D, respectively). Slight iridodonesis is observed

CEL is commonly observed in connective tissue disorders and other conditions that lead to the dislocation of the lens from its natural position. The findings of the current analysis of clinical features and multimodal biometric parameters in children with CEL and myopia demonstrate that AL/CR may be a new potential predictor for the identification of lens dislocation in children, especially for the differential diagnosis with myopia. Lens subluxation was observed after dilatation with compound topicamide, and results of eye examinations support the findings of the current study in terms of AL/CR changes in ocular parameters (Fig. 3). Therefore, the abnormal biological parameters may alert the ophthalmologists to a child with not only myopia but also CEL in a non-mydriasis setting.

In the current study, we matched the CEL group and control group in terms of the SE, and found that changes in ocular biological parameters, especially AL/CR < 3.024, were used to initially screen for occult lens subluxation, while avoiding missed diagnosis.

### SE and AL

Lenticular and axial myopia are the two components of myopia in CEL. Lenticular myopia may result from increasing curvature of the lens (spherophakia) due to the degeneration and relaxation of zonular fibers [6]. Mutations in FBN1 or other genes may also result in axial myopia owing to enlargement of the eyeball, and causing a longer AL [18]. Moreover, defocus and vision deprivation caused by lens displacement can cause pathological AL development, resulting in moderate-to-high myopia [9]. In the current study, the average SE was − 6.25(-8.719~-4.688) D in 3–7-year-old children with CEL, which was far greater than the Ghent-2 criteria for MFS.

Previous studies have reported that MFS patients AL was increased [13, 14, 19–21]. Wang et al. found that MFS patients had longer AL (24.71 ± 1.93 vs. 24.00 ± 1.54, p = 0.049) compared with non-MFS patients in a group of 3–6-year-olds [21]. Mulvihill et al. demonstrated that ocular AL was significantly increased in individuals with homocystinuria and lens dislocations [22]. However, the results of the current study showed that the AL in CEL patients was significantly shorter than in myopic patients. In our study, the SE in the two groups was identical, we propose that the main cause of myopia in CEL may be the displacement and deformation of the lens. Although we did not set healthy eyes as a control group, the AL of children with CEL still increased compared to previous studies [23, 24].

### Corneal curvature

A number of publications have demonstrated that CC in MFS eyes was flatter than that in non-MFS eyes, and some have proposed a threshold for corneal power of < 41.5 D [9, 13, 15, 25]. It has been suggested that the flattened corneas observed in CEL eyes are due to the increased dimension of the whole eyeball caused by fibrillin gene mutations in the sclera and cornea [9, 15]. Another explanation is that the lower corneal power compensates for the defocus of vision caused by axial growth in myopia [25, 26]. The present study revealed that the average Kmed in the CEL group was dramatically flatter than that in the myopic group. In the CEL group, 71% of eyes were flattened below 41.915 D versus only 7% in the myopic group. However, the AL in the CEL group was significantly shorter than that in the myopic group. Our study demonstrated that the decrease in CC in CEL may not be explained by the concerted mechanism alone and that total ocular enlargement is the major cause.

### Astigmatism

#### CA

Most studies have indicated that eyes with MFS and ectopia lentis have higher CA [13, 27]. The pathological changes in the cornea might be due to fibrillin gene mutations and associated corneal underdevelopment, a mechanism similar to the one that causes zonular instability and ectopia lentis [27]. However, Sultan et al. showed that in their control group, the mean astigmatism in the 3.0-mm central corneal zone (0.78 ± 1.4 D) was not significantly different from that in the MFS group (1.09 ± 0.87 D) [15]. Wang et al. found no significant difference in CA (1.79 ± 1.13 vs. 1.92 ± 1.07 D, p = 0.584) between MFS and non-MFS patients [21]. In our study, we found that the CA was lower in the CEL group than in the myopic group. In our opinion, the reason may be that our control group had moderate-to-high myopia. Touzeau et al. reported a mean CA of + 0.92 D×91.3° in the high myopic group and + 0.65 D×89.3° in the control group (p < 0.05) [28]. Their study indicated that the correlation with AL was significant for SE and the corneal cylinder. In the present study, the AL in the CEL group was shorter, and the CA was correspondingly lower. Therefore, the CA in the myopic group was higher than that in the CEL group, which might have increased to that of normal eyes, according to previous studies [13, 27].

#### CA and RA

IA and CA are the two components of RA, and CA is critical in normal eyes [29]. In our study, there was a significant difference in magnitude and axis between RA and CA in the CEL group and control groups. The results indicate that ectopia lentis is an important factor leading to internal astigmatism and, therefore, increased RA. Therefore, we should be aware of the possibility of ectopia lentis when a significant difference is found between the RA and CA.

### AL/CR

Many types of research studies have demonstrated that UBM is of great value in diagnosing lens subluxation and may be invaluable in surgical planning and therapeutic management [12, 30]. The implementation of UBM in children is more difficult than in adults because some procedures are invasive and cause discomfort in children, and additionally, they require the cooperation of the patient. Therefore, researchers have been trying to explore more effective diagnostic methods for lens subluxation in children. Extensive evidence indicates that, for emmetropic participants, a high AL/CR ratio (> 3.0) may serve as a risk factor for the development of myopia [31]. Previous studies have reported that the AL/CR was significantly larger in myopic eyes compared with nonmyopic eyes [32]. Chen et al. proposed that axial length / total corneal refractive power is a potential diagnostic factor that can be used for the early diagnosis of MFS [33]. Wang et al. showed that there was no significant difference in AL/CR (3.03 ± 0.29 vs. 3.01 ± 0.29, p = 0.993) between MFS and non-MFS patients [21]. The results of the current study showed that the AL/CR of CEL group were lower compared with the myopia group. Axial shortening and corneal flattening might have resulted in the reduction of AL/CR in the CEL group. Results from Wang et al. indicate that axial elongation may not be the main cause of myopia in CEL eyes [21].

### ROC

Luebke et al. reported that Kmax (area under the ROC 0.82, cut-off 41.36 D) provided the strongest effect for differentiation between the MFS and non-MFS groups [25]. Wang et al. found that the area under the ROC was 0.761 for AL, 0.736 for Kmed, and 0.713 for central corneal thickness in the MFS group compared to the non-MFS group [21]. They further indicated that a Kmed of 41.36 D combined with a central corneal thickness of 537.32 mm was the optimal cut-off point (sensitivity 89.8%, specificity 68.7%). The shorter AL, lower Kmed, and longer CR led to decreased AL/CR in CEL patients. AL/CR (area under the ROC 0.960, cut-off 3.024, sensitivity 92.90%, specificity 88.30%) could be used as a screening tool for the differential diagnosis of CEL and myopia. Compared to previous reports, this study suggests a highly specific, easy to master and convenient for use in the clinic [22, 26]. Our method shows additional advantages in the differential diagnosis of CEL, especially when SE < -6.00 D.

This study had several limitations. First, since it was not possible to conduct genetic testing for all children with CEL, a definitive diagnosis, such as MFS or homocystinuria, could not be obtained. Second, because the chosen age range was only 3–7 years, and some children did not cooperate with eye examination, a relatively small number of CEL patients were included, and thus, bias cannot be ruled out. Lastly, this was a retrospective study, and AL, which is a crucial parameter for eyeball development, can be influenced by many factors such as age and genetics.

## Conclusion

In conclusion, this study identified distinctive characteristics of ocular biological parameters of CEL in patients with moderate-to-high myopia, including shorter AL, flatter CC. Further analysis indicated that an AL/CR < 3.024 may be the most sensitive and specific parameter for use in screening for the differential diagnosis of CEL and myopic children.

## Data Availability

The data are available from the corresponding authors upon reasonable request.

## Abbreviations

CEL: Congenital ectopia lentis
AL: Axial length
CC: Corneal curvature
RA: Refractive astigmatism
CA: Corneal astigmatism
AL/CR: Axial length-corneal radius ratio
Kmed: Mean keratometry
Kmax: Maximum keratometry
Kmin: Minimum keratometry
ROC: Receiver operating characteristic
AUC: Area under the curve
